# Complete Genome Sequence of Lactobacillus helveticus VHProbi Y21, Isolated from Kimchi

**DOI:** 10.1128/mra.00941-22

**Published:** 2022-12-01

**Authors:** Hongchang Cui, Shumin Cheng, Zhi Duan

**Affiliations:** a Qingdao Vland Biotech, Inc., Qingdao, China; Loyola University Chicago

## Abstract

The probiotic strain Lactobacillus helveticus VHProbi Y21 confers beneficial effects in the prevention and treatment of acne. Here, we report the whole-genome sequence of this bacterium, which contains a chromosome and two plasmids, with a GC content of 37.52%.

## ANNOUNCEMENT

Spontaneous kimchi fermentation leads to the growth of lactic acid bacteria, which results in variations in the taste and sensory characteristics of kimchi products. In this study, we present the complete genome sequence of a bacterium that was isolated from kimchi and has the ability to produce propionic acid. A 1-mL sample of kimchi soup collected from homemade kimchi in Yanji city, China, was serially diluted in saline solution, and the 10^−9^ dilution was spread on a de-Man-Rogosa-Sharpe (MRS) agar plate. The inoculum was incubated at 37°C for 72 h under anaerobic conditions to enrich the bacteria. A colony named VHProbi Y21 was picked up and identified as the species Lactobacillus helveticus using 16S rRNA gene sequencing, as described by Estifanos et al. (1). A combined strategy with both Illumina HiSeq 2500 and Pacific Biosciences (PacBio) RS II platforms was used to sequence the genome of this strain. The bacterium was cultured using the same method as for enrichment to extract DNA. Total DNA was extracted using the Wizard genomic DNA purification kit (Promega) for Illumina and PacBio library preparation. DNA samples were sheared into ~350-bp fragments and then used for Illumina library preparation with the NEXTflex rapid DNA-sequencing kit. The library was used for paired-end Illumina sequencing (2 × 150 bp). Meanwhile, DNA samples was centrifuged in g-TUBES (Covaris, Woburn, MA) to obtain fragments. Then, the fragments were purified, end paired, and ligated with SMRTbell adapters. A ~10-kb insert library was used for PacBio RS II sequencing. Finally, 10,386,467 raw reads and 8,764,210 clean reads were generated using the Illumina sequencing platform, yielding a coverage depth of 724-fold; 554,668 raw reads (*N*_50_, 10,925 bp) were generated using the PacBio platform. The Illumina raw reads were quality filtered with Sickle v1.33 (https://github.com/najoshi/sickle). The PacBio raw reads were processed using single-molecule real-time (SMRT) Analysis v2.3.0 (2). The clean short and long reads were then coassembled to obtain the complete genome using Unicycler v0.4.8 (3). Pilon was used to polish the assembly results using the short-read alignments (4). If there was an overlap at the two ends of the assembly, then one end of the overlap was cut off to loop the sequence. The final assembly generated a circular chromosome and two circular plasmids. Glimmer v3.02 (5), tRNAscan-SE v2.0 (6), and barrnap v0.9 (https://github.com/tseemann/barrnap) were used to predict the protein-coding genes, tRNA genes, and rRNA genes, respectively. Genome annotations were performed using the NCBI Prokaryotic Genomic Annotation Pipeline (PGAP) v6.2 (7). Default parameters were used for all software unless otherwise specified.

The complete genome sequence of L. helveticus VHProbi Y21 comprises 2,171,060 bp in a circular chromosome and two circular plasmids, with a GC content of 37.52%. There were 1,952 protein-coding genes, 12 rRNA genes, 63 tRNA genes, 3 noncoding RNA genes, and 188 pseudogenes in the genome. The characteristics of the genome are shown in Table 1.

**TABLE 1:** Characteristics of the whole-genome sequence

Genomic element	Type	No. of genes	Replicon	GenBank accession no.
Chromosome	Circular	1,952		CP103857
pAY21	Circular	29	WP_012552803	CP103858
pBY21	Circular	18	WP_003712525	CP103859

### Data availability.

The whole-genome sequence of L. helveticus VHProbi Y21 has been deposited in GenBank with accession numbers CP103857, CP103858, and CP103859, SRA accession numbers SRR21166832 and SRR21166831, BioProject accession number PRJNA871029, and BioSample accession number SAMN30399899.
